# The Medical Department of the University of Buffalo

**Published:** 1891-04

**Authors:** 


### THE MEDICAL DEPARTMENT OF THE UNIVERSITY OF BUFFALO

Held its forty-fifth annual commencement at Music Hall, on Tuesday evening, March 24, 1891. The graduating class in medicine numbered seventy, and that in pharmacy thirteen, making a total of eighty-three. Hon. Jacob Stern addressed the Alumni Association, and Dr. John Wyeth, of New York, delivered the usual charge to the graduates. We go to press too early to give more than this brief notice of this interesting occasion, but hope to mention details next month.

The seventh annual announcement of the Spring course shows a steady growth in its usefulness and popularity. Several new courses have been added to the curriculum and will, undoubtedly, add to its interest. Dr. Edward Clark will give a course of lectures on Diseases of the Rectum. We congratulate the college on securing the services of so able a teacher in the department that he has chosen as his specialty. Dr. Grant will give a clinical course on Diseases of the Eye, and will, undoubtedly, prove the wisdom of the Faculty in choosing him.

Professor Park will give a course that will prove of great interest and value on Experimental Surgery, using living animals for purposes of investigation in this line.

Dr. Bergtold will lecture on Minor Surgery and Bandaging, thus giving practical instruction in a department that is often slighted.

Dr. Benedict will lecture on Prescription Writing, giving, in addition to the necessary form, a number of useful prescriptions and therapeutic hints.

Dr. Crockett will take up the subject of Venereal Diseases in Women, a subject that is of especial interest from the effect that these diseases have on the future condition of the pelvic and abdominal organs.

The other lectures will be as during last year, except that Dr. Rochester's clinic on Physical Diagnosis will be held at the General Hospital instead of the Fitch Dispensary, and that he will give the general medical clinics Wednesday and Saturday morning, in place of Dr. Hopkins.

The course promises to be one of unusual interest, and we presume the recent graduates and advanced students will attend it.

